# Human cleaving embryos enable robust homozygotic nucleotide substitutions by base editors

**DOI:** 10.1186/s13059-019-1703-6

**Published:** 2019-05-22

**Authors:** Meiling Zhang, Changyang Zhou, Yu Wei, Chunlong Xu, Hong Pan, Wenqin Ying, Yidi Sun, Yun Sun, Qingquan Xiao, Ning Yao, Wanxia Zhong, Yun Li, Keliang Wu, Gao Yuan, Shoukhrat Mitalipov, Zi-jiang Chen, Hui Yang

**Affiliations:** 1 Center for Reproductive Medicine, Ren Ji Hospital, School of Medicine, Shanghai Jiao Tong University, Shanghai, China; 2 Shanghai Key Laboratory for Assisted Reproduction and Reproductive Genetics, Shanghai, 200127 China; 3 Institute of Neuroscience, State Key Laboratory of Neuroscience, Key Laboratory of Primate Neurobiology, CAS Center for Excellence in Brain Science and Intelligence Technology, Shanghai Institutes for Biological Sciences, Chinese Academy of Sciences, Shanghai, 200031 China; 4 Center for Embryonic Cell and Gene Therapy, Oregon Health & Science University, 3303 Southwest, Bond Avenue, Portland, OR 97239 USA; 5 Center for Reproductive Medicine, Shandong Provincial Hospital Affiliated to Shandong University, Jinan, Shandong China; 6 National Research Center for Assisted Reproductive Technology and Reproductive Genetics, Jinan, China; 7 The Key laboratory for Reproductive Endocrinology of Ministry of Education, Jinan, 250021 Shandong China; 8 College of Life Sciences, University of Chinese Academy of Sciences, Beijing, 100049 China; 9 Key Lab of Computational Biology, CAS-MPG Partner Institute for Computational Biology, Shanghai Institutes for Biological Sciences, Chinese Academy of Sciences, Shanghai, 200031 China

**Keywords:** Base editing, Human cleaving embryos, Homozygotic nucleotide substitution

## Abstract

Base editing installs a precise nucleotide change in specific gene loci without causing a double-strand break. Its efficiency in human embryos is generally low, limiting its utility in functional genetic studies. Here, we report that injecting base editors into human cleaving two-cell and four-cell embryos results in much higher (up to 13-fold) homozygotic nucleotide substitution efficiency as opposed to MII oocytes or zygotes. Furthermore, as a proof-of-principle study, a point mutation can be efficiently corrected by our method. Our study indicates that human cleaving embryos provide an efficient base editing window for robust gene disruption and correction.

**Electronic supplementary material:**

The online version of this article (10.1186/s13059-019-1703-6) contains supplementary material, which is available to authorized users.

## Background

Base editors, enabling single nucleotide conversion without causing double-strand breaks, have been successfully applied for base correction in mouse and human embryos [1–5]. In contrast to the mouse, base-editing efficiency in human embryos is generally low (below 30%) that frequently leads to mosaicism and limits the utility of current base editing methods for gene functional study in human embryos (Additional file 1). Several species-specific differences in early embryonic development may account for low efficiency of BEs in human embryos. Here, we investigate whether injecting base editors into human embryos at different stages has an influence on base-editing efficiency.

## Results and discussion

**Fig. 1:**
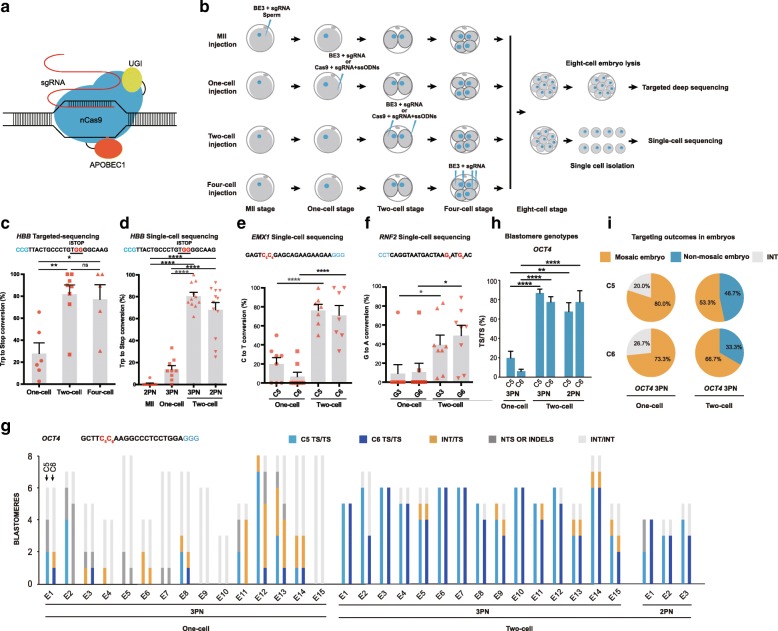
Improved base-editing efficiency in human cleaving embryos compared with MII oocytes and zygotes. **a** Experiment design. Different reagent mixtures were injected into MII oocytes, one-cell, two-cell, or four-cell stage embryos. Embryos were cultured to the eight-cell stage and used for targeted deep sequencing or single-cell sequencing. **b** Schematics of base editor components and working principle. **c** Targeted deep sequencing analysis of embryos injected with BE3 targeting *HBB* locus at one-cell, two-cell, or four-cell stage. Percentage of the total reads with targeted *Trp* codon to stop codon conversion on the *HBB* locus. SgRNA and PAM sequences are shown in black and blue, respectively. BE3-mediated nucleotide substitutions are shown in red. iSTOP, induction of stop codon. **d** Single-cell sequencing analysis of embryos injected with BE3 targeting *HBB* locus at MII, one-cell, two-cell, or four-cell stage. Percentage of alleles with targeted C>T conversions on the *HBB* is shown. 2PN, two pronuclei; 3PN, three pronuclei. **e**, **f** Single-cell analysis of embryos injected with BE3 at one-cell or two-cell stage targeting *EMX1* (**e**) and *RNF2* (**f**) loci. Percentage of alleles with targeted C>T or G>A conversions is shown. **g** Blastomere genotyping results of embryos injected with BE3 targeting *OCT4* locus. TS, targeted substitution; NTS, non-targeted substitution; INT, intact. **h**, **i** Homozygotic on-target efficiency at blastomere (**h**) and embryo (**i**) level respectively with BE3 targeting *OCT4* locus. Each data point represents an individual embryo. Results are presented as mean ± SEM. *P < 0.05, **P < 0.01, ***P < 0.001, ****P < 0.0001, unpaired Student’s *t* test. ns, not significant

To test the base-editing system in human embryos, we initially injected BE3 mRNA and sgRNA into one-cell embryo (zygote stage) (Fig. 1a, b and Additional file 1: Supplementary Methods) to induce G>A conversions (g.97G>A, G_8_; g.98G>A, G_7_) in exon 1 of *β-globin* (*HBB*) gene (Fig. 1a–c). BE3 mRNA and sgRNAs were co-injected into the cytoplasm of 3PN zygotes approximately 24 h post fertilization (one-cell stage) (Fig. 1b). Injected zygotes were cultured to the eight-cell stage and used for on-target deep sequencing analysis (Additional file 5: Table S3. Primers used in the study). Although expected Trp16 to Stop conversions (g.97G > A or g.98G > A) in the *HBB* locus were observed in some blastomeres of all injected embryos (*n* = 6), base-editing frequency was relatively low with high mosaicism by one-cell injection (27.8 ± 9.7%; Fig. 1c, Additional file 2: Figure S1a, and Additional file 3: Table S1).

The onset of zygotic gene activation (ZGA) in human embryos (four- to eight-cell stage) is typically later than that of mouse embryos (two-cell stage) [6, 7]. Therefore, we decided to test the conversion efficiency in cleaving human embryos by injecting BE3 mRNA and corresponding sgRNA into each blastomere of the two-cell or four-cell stage embryos (Fig. 1a) and measuring outcomes in eight-cell embryos. Remarkably, the efficiency of targeted G>A conversions was greatly increased and reached 82.6 ± 8.7% when injected into two-cell and 77.2 ± 13.3% into four-cell embryos in contrast with low efficiency in one-cell embryo (Fig. 1c, Additional file 2: Figure S1a, and Additional file 3: Table S1). Furthermore, we corroborated this finding by targeted g.22909C>T (C_5_) and g.22910C>T (C_6_) conversions in the exon 3 of *EMX1* gene and confirmed a significant increase in base-editing efficiency when treated cleaving human embryos as opposed to zygotes (Additional file 2: Figure S1b,c and Additional file 3: Table S1). We also used single-blastomere sequencing to analyze each cell of the multicellular embryo, which allows us to define the allelic targeting profile of each blastomere. After BE3 injection, each blastomere from the eight-cell embryos was isolated and individually sequenced (Fig. 1b). The efficiency of base editing at allelic level obtained from single-blastomere sequencing was consistent with that of deep sequencing analysis in *HBB*, confirming higher efficiency in two-cell injection than that in one-cell injection (80.50 ± 3.43% in two-cell versus 13.84 ± 3.33% in one-cell injection) (Fig. 1d and Additional file 4: Table S2). We also tested the base-editing efficiency in diploid embryos (2PN) at *HBB* locus and found similar results with 3PN ones (Fig. 1d; Additional file 4: Table S2; Additional file 5). It has been reported most zygotes had completed S phase of the cell cycle and DNA replication and likely produced four alleles for targeting, leading to high mosaicism [8, 9]. Therefore, we co-injected BE3 into MII oocytes with sperm during fertilization by intracytoplasmic sperm injection (ICSI) (Fig. 1b). Unexpectedly, MII oocyte injection resulted in much a lower base-editing frequency (0.63%) compared to pronuclear stage zygotes (13.8%) (Fig. 1d and Additional file 4: Table S2).

Though two-cell injection improved on-target efficiency compared with one-cell injection, indels or non-target substitution frequency stayed similarly low with less than 5% in both stages (Additional file 2: Figure S1e and Additional file 4: Table S2). Moreover, development competency of treated embryos to the eight-cell stage for *HBB* locus was not affected and comparable to uninjected control group (Additional file 2: Figure S1f). With experiments on more loci, we could also achieve the improved base-editing efficiency by two-cell injection at *EMX1*, *RNF2*, and *OCT4* loci (Fig. 1b–d, Additional file 2: Figure S1d, and Additional file 4: Table S2). Besides BE3-mediated C>T and G>A conversion, we additionally examined the efficiency of A>G and T>C conversions in cleaving human embryos by ABE system targeting three separate genomic loci, site 2, site 4, and site 6 [10]. Like the results in BE3 experiments, injection of ABE mRNA and corresponding sgRNA into two-cell human embryos resulted in significantly higher A>G conversions than in zygotes (Additional file 2: Figure S2a-c, Figure S3a-c and Additional file 4: Table S2).

After higher base-editing efficiency in cleaving embryos was verified at the allelic level, we further analyzed the homozygotic targeting efficiency in each blastomere. In this regard, we chose *OCT4* locus at the exon 1 to investigate the simultaneous induction of g.187C>T (C_5_) and g.188C>T (C_6_) conversions at three parental alleles by injecting BE3 into human two-cell embryos derived from 3PN zygotes (Fig. 1g). Single-blastomere analysis revealed that 87.4% (76 out of 87 blastomeres) of blastomeres carried desired C>T substitutions at the g.187C locus and 78.2% (68 out of 87 blastomeres) at the g.188C position in all three alleles (tri-allelic base substitutions) (Fig. 1g, h and Additional file 4: Table S2). By contrast, only 22.3% and 6.4% of blastomeres derived from conventional zygote injection carried g.187C>T and g.188C>T tri-allelic base substitutions, respectively (Fig. 1g, h and Additional file 4: Table S2). Besides 3PN embryos, we also targeted the same *OCT4* locus in two-cell embryos derived from normally fertilized (2PN) zygotes. The percentage of homozygotic targeted blastomere was 68.3% for the g.187C locus and 78.3% for the g.188C site, comparable to those derived from abnormally fertilized 3PN embryos (Fig. 1g, h and Additional file 4: Table S2). Remarkably, 5 out of the total 15 two-cell injected embryos derived from 3PN zygotes carried homozygous C>T substitutions at both g.187C and g.188C loci, whereas none of one-cell injected embryos have such complete editing in each blastomere (Fig. 1i).

In addition to *OCT4* locus, we also found significant improvement of homozygotic conversion efficiency in other loci including *HBB*, *EMX1* by two-cell injection compared to one-cell injection (Additional file 2: Figure S4a-d and Additional file 4: Table S2). These results indicate that base editors injection in cleaving embryos could efficiently induce nucleotide substitutions simultaneously in all parental alleles in a single blastomere, suggesting potential applications to interrogate the causality between homozygous point mutations and corresponding phenotype in human embryos.

**Fig. 2:**
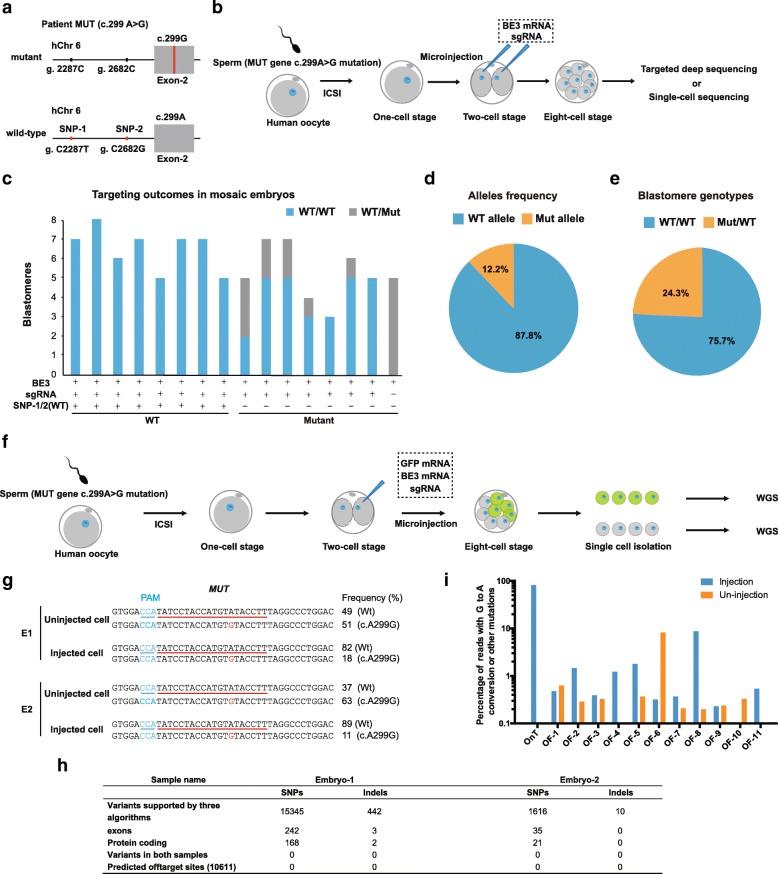
Correction of a pathogenic heterozygous mutation in human embryos with base editors. **a** Diagram of *MUT* c.299 A>G mutation locus for a male patient. Exon is labeled with a gray box; c.299A>G mutation site is indicated with a red line. In addition, mutant and wild-type alleles of this patient can be distinguished by two adjacent neutral SNPs. **b** Experimental diagram showing BE3-medicated gene correction in human embryos. Sperm from a heterozygous patient was used to fertilize the oocytes. BE3 mRNA and *MUT* sgRNA were co-injected into each blastomere of the two-cell embryos 36 h after fertilization. Embryos were cultured to eight-cell embryos and used for targeted deep sequencing or single-cell sequencing. **c** Blastomere genotyping results in injected embryos. WT, wild-type; Mut, mutant c.299A>G. **d**, **e** Allele frequency and blastomere genotypes in BE3-treated heterozygous embryos. **f** Schematic of off-targeting analysis using whole genome sequencing of BE3-treated embryos. BE3 mRNA, OCT4 sgRNAs, and GFP mRNA were co-injected into one blastomere of two-cell embryos whereas another blastomere left uninjected. When embryos developed to the eight-cell stage, GFP-positive and negative blastomeres were separated and analyzed by WGS. **g** Alignments and percentage of mutant and corrected sequences from embryos injected with BE3 mRNA and *MUT* sgRNA. The target sequence is underlined. PAM site and substitutions are shown in blue and red, respectively. The column on the right indicates frequencies of mutant alleles. WT, wild-type. **h** Variant calling results revealing no off-target event detected by WGS. Indels, insertion or deletion; SNV, single nucleotide variants. **i** Targeted deep sequencing analysis of on-target and 11 potential off-target loci in *MUT* c.299 A>G mutant embryos with or without base editing

We next tested whether base editors could be used for correcting point mutation and interrogating causality between mutations and corresponding phenotypes in early human embryonic development. As a proof-of-concept study, we chose a previously identified c.299A>G mutation in *MUT* gene encoding methylmalonyl CoA mutase for the correction experiment. Homozygous c.299A>G substitution in *MUT* leads to methylmalonic acidemia, a condition characterized by feeding difficulties, developmental delay, and long-term health problems [11]. We identified an adult male with the heterozygous c.299A>G (g.4133A>G) mutation, and he consented to donate a semen sample. In contrast to mutant c.299A>G allele, normal wild-type gene carried two linked neutral SNPs (NC_000006.12, g.2259C>T; NC_000006.12, g.2654C>G) (Fig. 2a and Additional file 2: Figure S5a, b).

We then fertilized in vitro matured MII oocytes with the carrier sperms and injected BE3 mRNA with sgRNA into two-cell embryos (Fig. 2b). Embryos were further cultured into the eight-cell stage and used for single-blastomere analysis. Original heterozygous mutant embryos (MUT^+/C.299A>G^) produced from the mutant sperm were identified and separated from wild-type (MUT^+/+^) embryos by the presence of the linked SNPs. In intact controls, 50% (8/16) of embryos were MUT^*+/+*^ while the other half (8/16) were MUT^+/C.299A>G^ (Additional file 2: Figure S5c and Additional file 4: Table S2). In embryos injected with BE3 mRNA and sgRNA, 10 out of total 15 (66.7%) were uniformly homozygous (MUT^*+/+*^), among which 2 embryos were fertilized by mutant sperm (MUT ^c.299A>G^) (Fig. 2c). The remaining 5 embryos (33.3%) were mosaic carrying 2 types of blastomeres, MUT^*+/+*^ and MUT^+/C.299A>G^ (Fig. 2c). However, in the 5 mosaic and 2 completely corrected mutant embryos, 87.6% of analyzed alleles were WT and 75.7% (28/35) of blastomeres became homozygous with only wild-type genotype of MUT^*+/+*^, indicating the proper correction of the mutant paternal allele with base editing (Fig. 2d, e). Furthermore, all BE3-treated embryos derived from the WT sperm were uniformly homozygous (MUT^*+/+*^) without any misconversions or indels indicating high specificity of base editing (Fig. 2c).

We next investigated if base editing induced any off-target alterations. To eliminate the differences in the genetic background between the gene-edited and control embryos, BE3, GFP mRNA, and sgRNA were co-injected into only one blastomere of two-cell embryos while leaving another one uninjected (Fig. 2f). Injected blastomeres were identified by GFP expression (GFP^+^) in eight-cell embryos. Whole genome sequencing (WGS) was performed on both GFP^+^ and GFP^−^ cells, and multiple variant-calling software pipelines were used to ensure reliable identification of indels and single nucleotide variants (SNVs) (Fig. 2g, h). In the results from two BE3-edited embryos analyzed, we found neither variants shared in two BE3-edited embryos nor variants in 10,611 predicted off-target sites (Fig. 2h). We also performed targeted deep sequencing to verify the top 11 predicted off-target sites and still found no evidence for off-target mutations (Fig. 2i and Additional file 4: Table S2).

In summary, we showed that the delivery of base editors into cleaving two-cell or four-cell human embryos resulted in much higher homozygotic nucleotide conversion rates, possibly due to more compact chromatin in human zygotes and massive RNA degradation event around zygote cleavage stage (Additional file 2: Figure S6a, b) [12]. A recent work reported the correction of a Marfan syndrome (MFS) pathogenic mutation in embryos by base editing [5]. However, the conclusions were untenable due to the low number of embryos and inadequate experimental design and data analysis (Additional file 2: Figure S7a, b). Notably, two recent studies have reported that BE3 generates substantial off-target mutations in mouse embryos and rice [13, 14]. However, no overlapped mutation was found in our study between any two individual embryos and very few of them located on exon, unlikely affecting the base editor application for gene function study during human embryonic development. Certainly, it will be highly desirable to explore and use base editors of high efficiency and fidelity for gene manipulation in human embryos in the future.

## Methods

### Retrieval of 3PN embryos during in vitro fertilization

The COCs were inseminated in 4-well plates with approximately 100,000 motile spermatozoa for each oocyte. Approximately 18–20 h after fertilization, we collect 3PN embryos for the experiment.

### Derivation of 2PN embryos by ICSI

Immature MI oocytes were collected from patients for IVF or ICSI treatment. MI oocytes were cultured in IVM medium in vitro for the first polar body extrusion by observation every 2 h. ICSI was performed 3 h after polar body extrusion.

### Injection of base editors into embryos

For one-cell injection, the mixture of BE3/ABE mRNA (100 ng/μl) and sgRNA (50 ng/μl) was injected into the cytoplasm of the zygotes 24 h after fertilization. For two-cell or four-cell injection, the mixture of BE3/ABE mRNA (100 ng/μl) and sgRNA (50 ng/μl) was injected into every blastomere of two-cell or four-cell embryos 36 or 44 h after fertilization, respectively.

### Single-blastomere sequencing analysis

Individual blastomeres were put into PCR tubes with 1.5 μl embryo lysis buffer and used for nest PCR. The PCR product was analyzed by Sanger sequencing to detect the efficiency of base editing.

### Statistical analysis

All statistical values were presented as mean ± SEM. Differences between datasets were considered to be significant at *P* value less than 0.05. All the statistic tests were conducted with Student *t* test unless otherwise stated.

## Additional files

Additional file 1: Supplementary methods. (DOCX 26 kb)

Additional file 2: **Figure S1.** Highly increased base-editing efficiency in cleaving human embryos compared with zygote. **Figure S2.** Improved base-editing efficiency in human cleaving embryos with ABEs. **Figure S3.** Frequency of indel mutations and off-targeted nucleotide substitutions in human embryos injected by base editors. **Figure S4.** Targeting homozygous loci in human embryos with base editors. **Figure S5.** Identification of SNPs in the patient distinguishing MUT c.299A>G from WT allele. **Figure S6.** Cleaving embryos have a higher level of GFP fluorescence than one-cell embryo 24 h post-mRNA injection. **Figure S7.** Comparison results of base editing in human embryos in two studies. (PDF 1771 kb)

Additional file 3: **Table S1.** Targeted deep sequencing results. (XLSX 17 kb)

Additional file 4: **Table S2.** Single-cell sequencing results. (XLSX 54 kb)

Additional file 5: **Table S3.** Primers used in the study. (XLSX 12 kb)

Additional file 6: Review history. (DOCX 33 kb)

## Abbreviations

2PN/3PN: Two/three pronuclei
ABE: Adenine base editor
BEs: Base editors
DSB: Double-strand break
ICSI: Intracytoplasmic sperm injection
IVF: In vitro fertilization
MFS: Marfan syndrome
MII: Metaphase II
SNP: Single nucleotide polymorphism
SNVs: Single nucleotide variants
UGI: UNG inhibitory protein
WGS: Whole genome sequencing
ZGA: Zygotic gene activation
