# Supplementary material for: Human cleaving embryos enable robust homozygotic nucleotide substitutions by base editors
Source: Genome Biol. 2019 May 22;20:101. doi: 10.1186/s13059-019-1703-6 (PMC6532253; doi:10.1186/s13059-019-1703-6)
Supplement: Supplementary file 1 — Supplementary methods. (DOCX 26 kb) [file 13059_2019_1703_MOESM1_ESM.docx]

Additional file 1: Supplementary methods.

**Injection of base editors into embryos**

For 1-cell injection, the mixture of BE3/ABE mRNA (100 ng/μl) and sgRNA (50 ng/μl) was injected into the cytoplasm of zygotes 24 h after fertilization using a FemtoJet microinjector (Eppendorf) with constant flow settings. For 2-cell or 4-cell injection, the mixture of BE3/ABE mRNA (100 ng/μl) and sgRNA (50 ng/μl) was injected into every blastomere of 2-cell or 4-cell embryos 36 or 44 h after fertilization respectively. The injected embryos were transferred into G1.5 media (Vitrolife, Sweden) until day 3 and transferred into G2 media (Vitrolife, Sweden) for later stage culture. All embryos were cultured in the condition of 37°C, 6% CO_2_, 5% O_2_, and 89% N_2_.

**Targeted Deep Sequencing**

Target sites and all the identified off-target sites were amplified from genomic DNA using Phanta Max Super-Fidelity DNA Polymerase (Vazyme Biotech Co., Ltd). The paired-end sequencing of PCR amplicons was performed by CloudHealth Genomics Co., Ltd using an HiSeq X Ten. A list of primers used in this study is shown in (Additional file 5: Table S3.). Quality control was performed on the raw sequencing data using fastQC (v0.11.3), qualified reads were subsequently demultiplexed using cutadapt with the PCR primers (v1.14). We next performed sequence alignment between the demultiplexed sequencing data with each of the on-target and off-target sites with blast (v2.2.29+), and then make mapping statistics using in-house scripts with Perl (v5.20.2) and R (v3.2.5).

**Single blastomere sequencing analysis**

Zonae pellucidae of 8-16 cell embryos were removed by Tyrode’s Solution (Sigma). Zona-free embryos were digested into single cells by 0.05% Trypsin-EDTA solution (Gibco). Individual blastomeres were put into PCR tubes with 1.5 μl embryo lysis buffer (0.1% Tween-20, 0.1% Triton X-100 and 4 μg/ml proteinase K) and incubated at 56°C for 30 min, heat inactivated at 95°C for 10min. PCR amplification was performed using nested primer sets and ExTaq PCR kit (ExTaq). First-round PCR program used was as follow: 95°C for 30s, 62°C for 30s, and 72°C for 1min, with a final extension at 72°C for 5min. Secondary PCR was performed using 0.5 μl PCR product and nested inner PCR primers, and carried out with the same program used in the first-round. The PCR product was analyzed by Sanger sequencing to detect efficiency of base editing. It was regarded as homozygous for wildtype or edited genotypes when sequencing results for each blastomere detected only one type of allele. In *MUT* gene correction experiment, two neutral SNP were used to distinguish between wildtype and mutant sperm fertilized embryos and only embryos carrying mutant gene were used for editing efficiency analysis. Therefore, among injected embryos carrying mutant genes, it was regarded as corrected when sequencing results for single blastomere detected only WT allele.

**Whole Genome Sequencing and off-target analysis**

Whole genome amplification of the embryos was performed using the PEPLO-g midi kit (Qiagen). Briefly, embryos were digested with acid Tyrode solution to remove the zona pellucide and transferred into PCR tubes containing reconstituted buffer D2 (7μl), and then incubated at 65°C for 10min, before the addition of top solution (3.5μl) and MDA master mix (40μl) and incubation at 30℃ for 8h. The DNA preparation was diluted with ddH_2_O (1:30), and 1μl of the diluted DNA was used for PCR analysis. Whole genome sequencing was carried out using Illumina HiSeq X Ten at mean coverages of 20X. Qualified reads were mapped to the human reference genome (hg19) by BWA (v0.7.12) with default parameters. All of the mapped data is available from the SRA under accession SRP136376. The BAM files were then sorted and PCR duplicates were marked using Picard ‘MarkDuplicates’. Mutect, Lofreq, and Strelka were run separately on the aligned BAM file of GFP+ cells for *de novo* SNV detection, with the GFP- sample in the same embryo as control. Whole genome *de novo* indels were detected using Mutect2, Scalpel, and Strelka in the same way[16-23]. For example, if the WT allele is G in a certain coordinate, the GFP+ cells carries A, and the GFP- cells carries G, then the mutant A will be called as a de novo mutation. While, if GFP- cells carries A, the mutant could not be identified. SNVs and indels supported by all the three algorithms were reserved for the following analysis, respectively. Besides, variants that located in low complexity regions including UCSC repeat regions and microsatellite sequences were filtered and removed from the following analysis [21]. To make functional annotations for the identified variants, we applied annovar (version 2016-02-01) on both the detected SNVs and indels using RefSeq database of mm10 [22].

Potential off-target sites of sgRNAs were predicted as previously reported (<http://www.rgenome.net/cas-offinder/>) [23]. We extracted all the off-target sites with no more than 8 mismatches and 2 DNA or RNA bulges for each sgRNA. We then mapped the non-synonymous SNVs and frameshift indels to the predicted off-targets by chromosome positions.

**Human oocyte retrieval and sperm preparation**

Before oocyte retrieval, all donors or patients had signed the informed consent of remaining gamete donation for research. Cumulus-corona oocyte complexes (COCs) were isolated from the follicle fluid promptly and precisely, and then cultured in G-IVF (Vitrolife, Sweden) for three hours. Semen samples were collected on day of oocyte retrieval by masturbation after 3−5 days of abstinence. Semen was kept for 30 minutes at 37°C for liquefaction, followed by a conventional density-gradient separation method. Briefly, after the second centrifugation, the pellet was usually overlaid with 0.5 ml of G-IVF and incubated for swim-up for 30 min. The supernatant was used for insemination.

**Retrieval of 3PN embryos during *in vitro* fertilization (IVF)**

The COCs were inseminated in 4-well plates with approximately 100,000 motile spermatozoa for each oocyte. Approximately 18-20h after fertilization, collect 3PN embryos for experiment. Sequential culture media from Vitrolife (G-IVF, G1, and G2; Sweden) were applied in all steps.

**Derivation of 2PN embryos by ICSI**

Immature MI oocytes were collected from patients for IVF or ICSI treatment. All oocytes were retrieved under informed consent from donor or patients. MI oocytes were cultured in IVM medium (TCM199 + 10%FBS + 10μg/ml Sodium pyruvate + 10μg/ml FSH + 5μg/ml LH + 1μg/ml E2+1nM melatonin) *in vitro* for the first polar body extrusion by observation every 2 hours. Three hours after polar body extrusion, ICSI was performed and embryos were cultured in time-lapse incubator (Vitrolife Embryoscope) at 37°C in 6% CO_2_, 5% O_2_, and 89% N_2_. These embryos were then cultured in G1 media (Vitrolife, Sweden) and 74h after fertilization were transferred into G2 media (Vitrolife, Sweden) for further culturing.

**Sperm cryopreservation and thawing**

Semen samples were collected by masturbation into sterile containers after 3–5 days of sexual abstinence and left to liquefy at 37 °C. Each complete semen sample was placed in a 15-ml centrifuge tube (BD, Falcon, USA), diluted 1:1 with yolk buffer (Irvine Scientific, Ca, USA) in a slow drop-wise manner, gently mixing it to form a homogeneous solution. This solution was equally aliquoted into 0.25-ml straw (Nunc International, Denmark), previously labeled with the individual ID of each sample. The 200-µl mixture of semen and cryoprotectant was aspirated into a 0.25-ml straw; the straw was then covered with an obturation and identification rod, placed at 4 cm (-170 ºC to −180 ºC) above the surface of the nitrogen vapor for 15 min and then directly transferred into LN2 for preservation. The straw was warmed in a water bath at 37 °C for 1 min. Then, the obturation and identification rod was pulled out and the solution lightly aspirated into a 1.5-ml Safe-Lock microcentrifuge tube that contained 1 ml pre-warmed GOMPS medium at 37 °C. We washed the straw with 1 ml prewarmed GOMPS medium at least twice. The mixture was subsequently centrifuged at 360 g for about 5 min. The supernatants were discarded, and the pellets were resuspended in 100 μl of sperm medium and kept at 37 °C until used for sperm motility evaluations and ICSI.

**Production of mRNA and sgRNA**

The pX330, pCMV-BE3 and pCMV-ABE were obtained from Addgene (Cat. No. 42230, Cat. No.73021, Cat. No. 107723). The pCMV-BE3/ABE plasmids were linearized with BbsI and used as the template for *in vitro* transcription using the mMESSAGE mMACHINE T7 Ultra kit (Life Technologies). T7 promoter and sgRNA target sequence were added to sgRNA template by PCR amplification of pX330, using primer listed in Table S3. The T7-sgRNA PCR product was purified and used as template for *in vitro* transcription using MEGA shortscript T7 Kit (Life Technologies). The sgRNA and BE3 mRNA were purified using the MEGAclear kit (Life Technologies) and eluted in RNase-free water. *In vitro* transcribed RNAs were aliquoted and stored at -80°C until use. All sgRNA used in the study were designed with additional G on the 5’ end of gRNA sequence (Additional file 5: Table S3).

**Embryonic GFP mRNA injection and fluorescence imaging analysis**

GFP mRNA was *in vitro* transcribed with the mMESSAGE mMACHINE T7 Ultra kit (Life Technologies). The same amount of GFP mRNA (50 ng/μl) was injected into 1-cell, 2-cell, and 4-cell human embryos via a FemtoJet microinjector (Eppendorf) with constant flow settings. The GFP fluorescent images were acquired using Olympus FV10I and analyzed with ImageJ software.

**Reference**

16. Cibulskis K, Lawrence MS, Carter SL, Sivachenko A, Jaffe D, Sougnez C, Gabriel S, Meyerson M, Lander ES, Getz G: Sensitive detection of somatic point mutations in impure and heterogeneous cancer samples. Nat Biotechnol 2013, 31:213-219.

17. Wilm A, Aw PP, Bertrand D, Yeo GH, Ong SH, Wong CH, Khor CC, Petric R, Hibberd ML, Nagarajan N: LoFreq: a sequence-quality aware, ultra-sensitive variant caller for uncovering cell-population heterogeneity from high-throughput sequencing datasets. Nucleic Acids Res 2012, 40:11189-11201.

18. Saunders CT, Wong WS, Swamy S, Becq J, Murray LJ, Cheetham RK: Strelka: accurate somatic small-variant calling from sequenced tumor-normal sample pairs. Bioinformatics 2012, 28:1811-1817.

19. Ye K, Schulz MH, Long Q, Apweiler R, Ning Z: Pindel: a pattern growth approach to detect break points of large deletions and medium sized insertions from paired-end short reads. Bioinformatics 2009, 25:2865-2871.

20. Narzisi G, O'Rawe JA, Iossifov I, Fang H, Lee YH, Wang Z, Wu Y, Lyon GJ, Wigler M, Schatz MC: Accurate de novo and transmitted indel detection in exome-capture data using microassembly. Nat Methods 2014, 11:1033-1036.

21. Iyer V, Shen B, Zhang W, Hodgkins A, Keane T, Huang X, Skarnes WC: Off-target mutations are rare in Cas9-modified mice. Nat Methods 2015, 12:479.

22. Wang K, Li M, Hakonarson H: ANNOVAR: functional annotation of genetic variants from high-throughput sequencing data. Nucleic Acids Res 2010, 38:e164.

23. Bae S, Park J, Kim JS: Cas-OFFinder: a fast and versatile algorithm that searches for potential off-target sites of Cas9 RNA-guided endonucleases. Bioinformatics 2014, 30:1473-1475.

24. Zhang M, Zhou C, Wei Y , Xu C , Pan H , Ying W, et al, Human cleaving embryos enable robust homozygotic nucleotide substitutions by base editors. Data sets. SRA. https://www.ncbi.nlm.nih.gov/sra/SRP136376 (2019).
