# Supplementary material for: Human cleaving embryos enable robust homozygotic nucleotide substitutions by base editors
Source: Genome Biol. 2019 May 22;20:101. doi: 10.1186/s13059-019-1703-6 (PMC6532253; doi:10.1186/s13059-019-1703-6)
Supplement: Supplementary file 2 — Figure S1. Highly increased base-editing efficiency in cleaving human embryos compared with zygote. Figure S2. Improved base-editing efficiency in human cleaving embryos with ABEs. Figure S3. Frequency of indel mutations and off-targeted nucleotide substitutions in human embryos injected by base editors. Figure S4. Targeting homozygous loci in human embryos with base editors. Figure S5. Identification of SNPs in the patient distinguishing MUT c.299A>G from WT allele. Figure S6. Cleaving embryos have a higher level of GFP fluorescence than one-cell embryo 24 h post-mRNA injection. Figure S7. Comparison results of base editing in human embryos in two studies. (PDF 1771 kb) [file 13059_2019_1703_MOESM2_ESM.pdf]

Figure S1

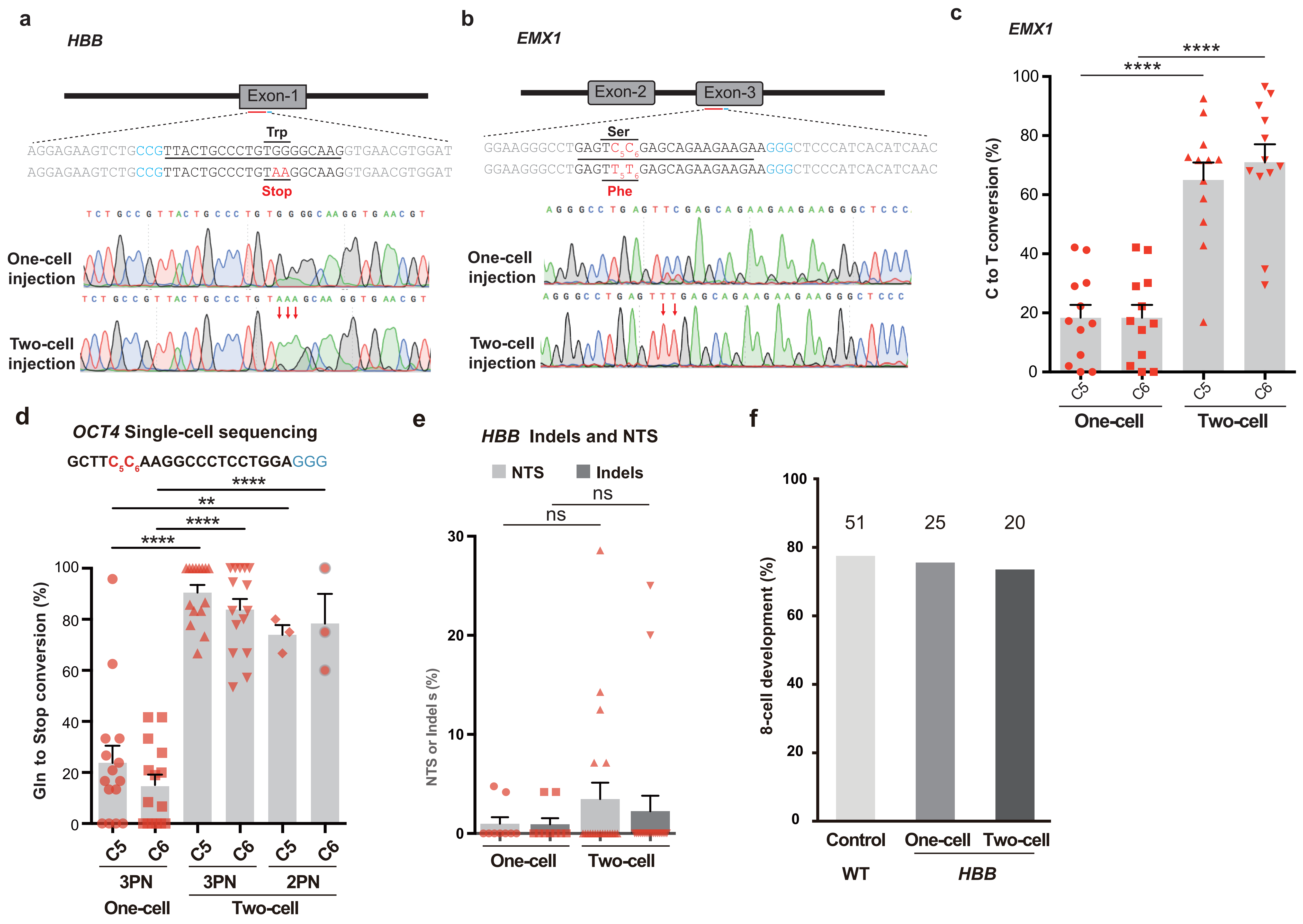
a
HBB
Exon-1
Trp
AGGAGAAGTCTGCCGTTACTGCCCTGTGGGGCAAGGTGAACGTGGAT
AGGAGAAGTCTGCCGTTACTGCCCTGTAAGGCAAGGTGAACGTGGAT
Stop
One-cell injection
Two-cell injection
b
EMX1
Exon-2
Exon-3
Ser
Phe
One-cell injection
Two-cell injection
c
EMX1
C to T conversion (%)
****
****
C5
C6
One-cell
Two-cell
d
OCT4 Single-cell sequencing
GCTTC5C6AAGGCCCTCCTGGAGGG
Gln to Stop conversion (%)
****
**
3PN
2PN
One-cell
Two-cell
e
HBB Indels and NTS
NTS
Indels
ns
NTS or Indels (%)
One-cell
Two-cell
f
8-cell development (%)
51
25
20
Control
One-cell
Two-cell
WT
HBB

Figure S2

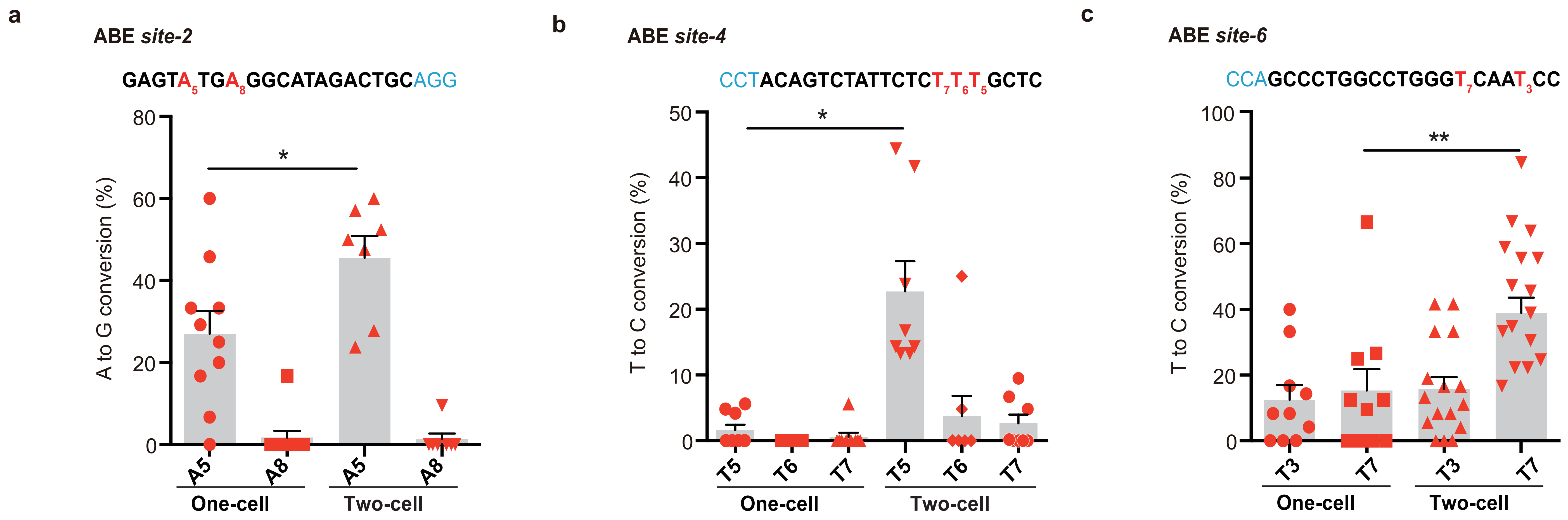

a
ABE site-2
GAGTA5TGA8GGCATAGACTGCAGG
A to G conversion (%)
80
60
40
20
0
*
A5
A8
A5
A8
One-cell
Two-cell
b
ABE site-4
CCTACAGTCTATTCTCT7T6T5GCTC
T to C conversion (%)
50
40
30
20
10
0
*
T5
T6
T7
T5
T6
T7
One-cell
Two-cell
c
ABE site-6
CCAGCCCTGGCCTGGGT7CAAT3CC
T to C conversion (%)
100
80
60
40
20
0
**
T3
T7
T3
T7
One-cell
Two-cell

Figure S3

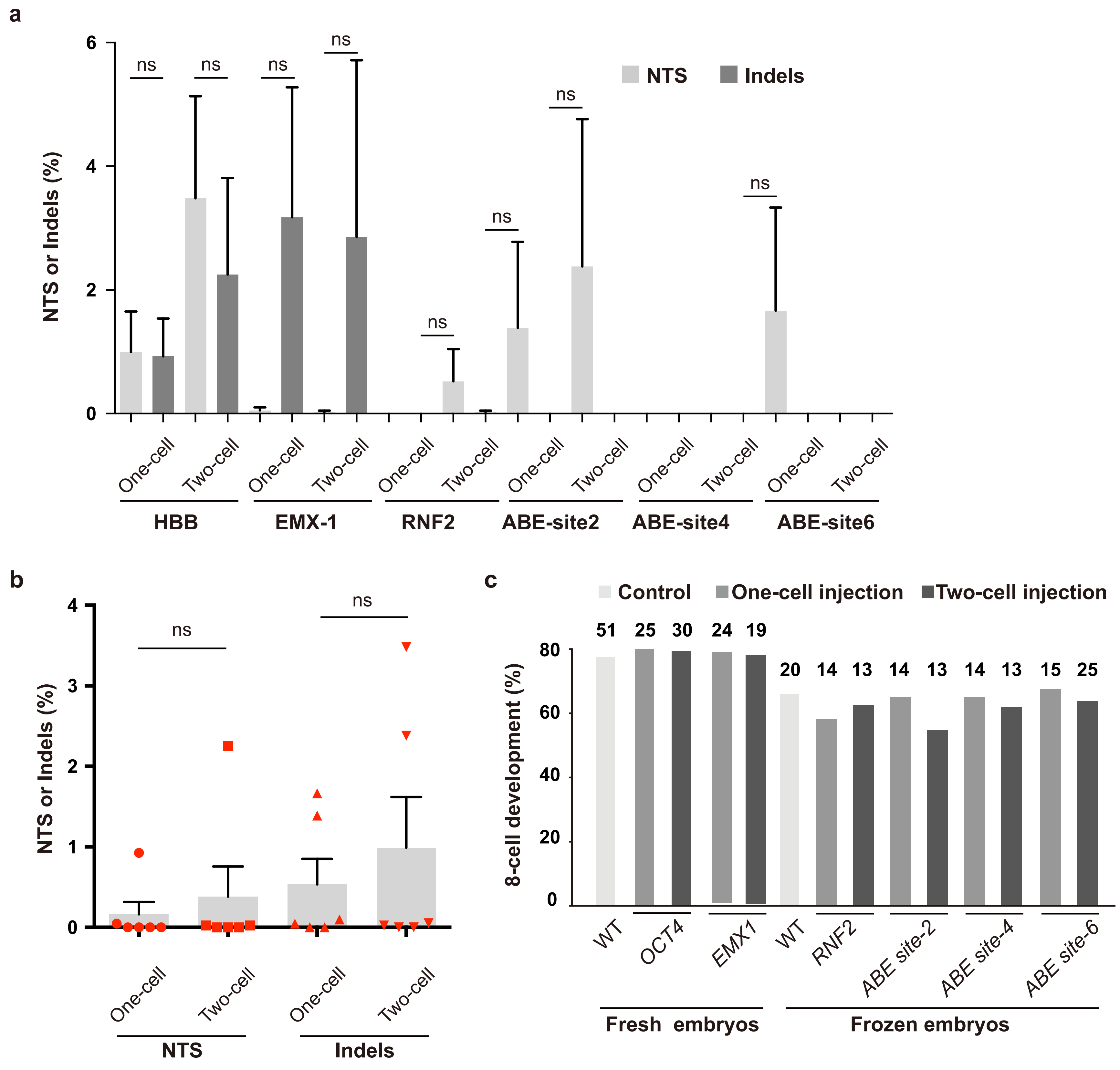
a
NTS
Indels
NTS or Indels (%)
ns
One-cell
Two-cell
HBB
EMX-1
RNF2
ABE-site2
ABE-site4
ABE-site6
b
NTS or Indels (%)
ns
One-cell
Two-cell
NTS
Indels
c
Control
One-cell injection
Two-cell injection
8-cell development (%)
51 25 30 24 19 20 14 13 14 13 14 13 15 25
WT
OCT4
EMX1
WT
RNF2
ABE site-2
ABE site-4
ABE site-6
Fresh embryos
Frozen embryos

Figure S4

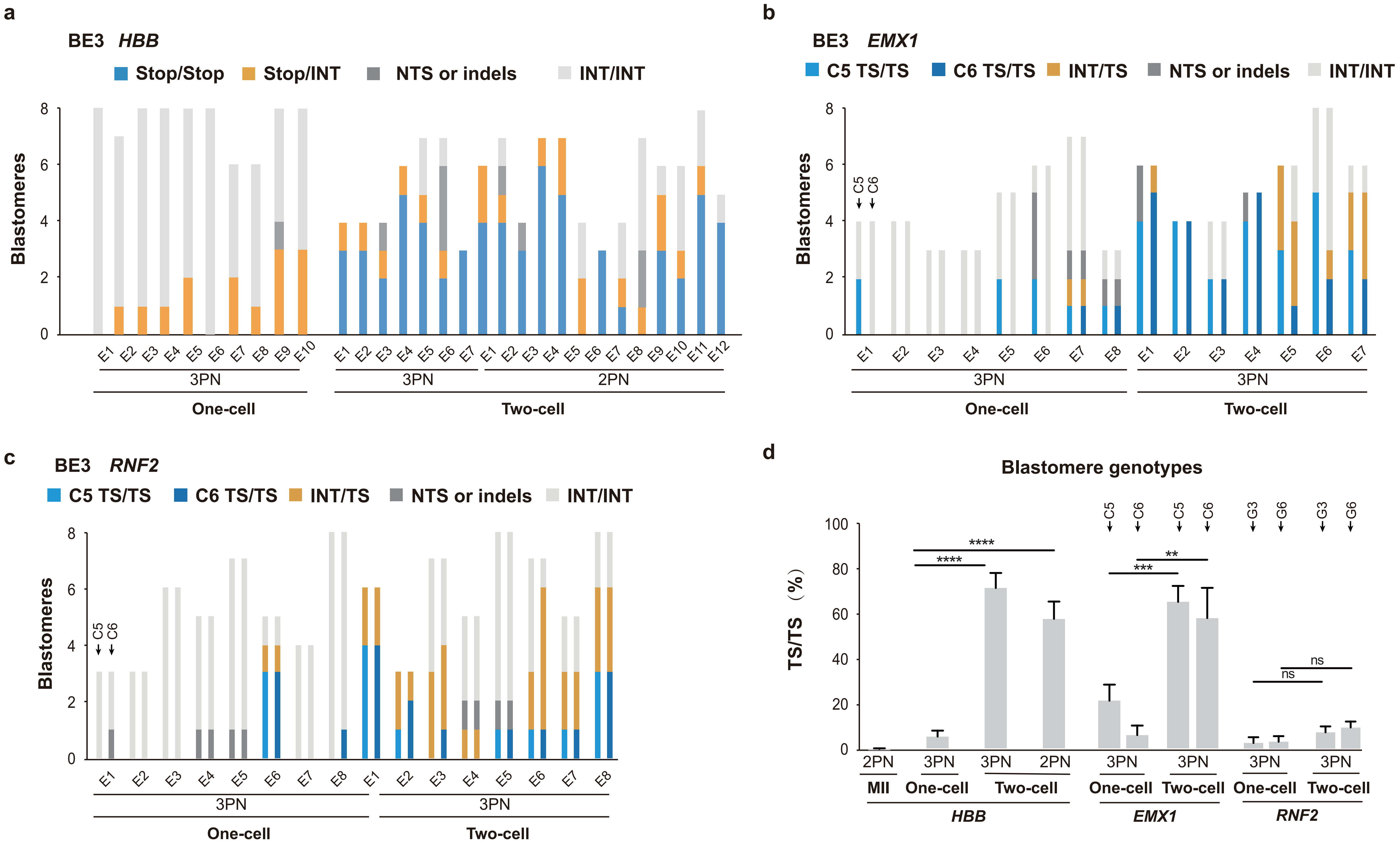
a
BE3 HBB
Stop/Stop
Stop/INT
NTS or indels
INT/INT
Blastomeres
0
2
4
6
8
E1 E2 E3 E4 E5 E6 E7 E8 E9 E10
E1 E2 E3 E4 E5 E6 E7
E1 E2 E3 E4 E5 E6 E7 E8 E9 E10 E11 E12
3PN
3PN
2PN
One-cell
Two-cell
b
BE3 EMX1
C5 TS/TS
C6 TS/TS
INT/TS
NTS or indels
INT/INT
Blastomeres
C5
C6
E1 E2 E3 E4 E5 E6 E7 E8
E1 E2 E3 E4 E5 E6 E7
3PN
3PN
One-cell
Two-cell
c
BE3 RNF2
C5 TS/TS
C6 TS/TS
INT/TS
NTS or indels
INT/INT
Blastomeres
C5
C6
E1 E2 E3 E4 E5 E6 E7 E8
E1 E2 E3 E4 E5 E6 E7 E8
3PN
3PN
One-cell
Two-cell
d
Blastomere genotypes
TS/TS (%)
0
20
40
60
80
100
****
****
***
**
ns
ns
C5 C6 C5 C6
G3 G6 G3 G6
2PN
3PN
3PN
2PN
3PN
3PN
3PN
3PN
MII
One-cell
Two-cell
One-cell
Two-cell
One-cell
Two-cell
HBB
EMX1
RNF2

Figure S5

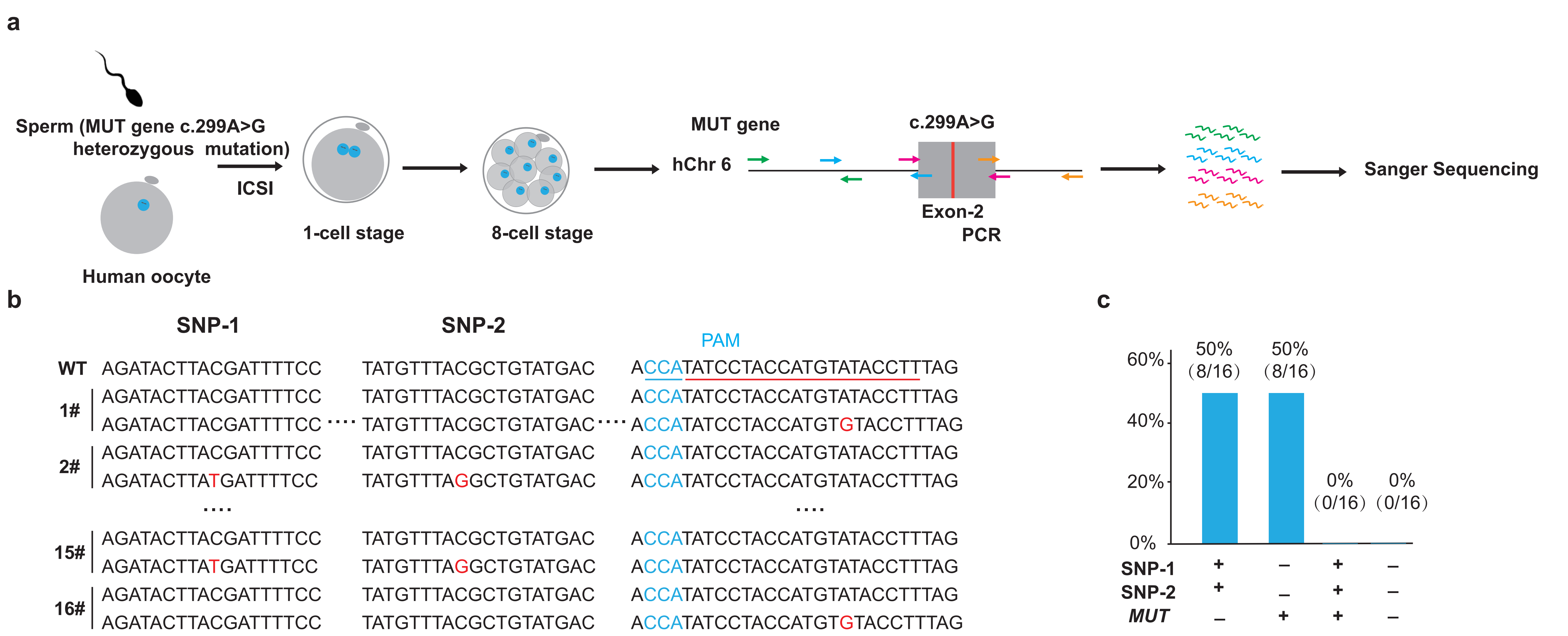
a
Sperm (MUT gene c.299A>G heterozygous mutation)
ICSI
Human oocyte
1-cell stage
8-cell stage
MUT gene
hChr 6
c.299A>G
Exon-2
PCR
Sanger Sequencing
b
SNP-1
SNP-2
PAM
WT AGATACTTACGATTTTCC TATGTTTACGCTGTATGAC ACCATATCCTACCATGTATACCTTTAG
1# AGATACTTACGATTTTCC TATGTTTACGCTGTATGAC ACCATATCCTACCATGTATACCTTTAG
AGATACTTACGATTTTCC TATGTTTACGCTGTATGAC ACCATATCCTACCATGTGTACCTTTAG
2# AGATACTTACGATTTTCC TATGTTTACGCTGTATGAC ACCATATCCTACCATGTATACCTTTAG
AGATACTTATGATTTTCC TATGTTTAGGCTGTATGAC ACCATATCCTACCATGTATACCTTTAG
15# AGATACTTACGATTTTCC TATGTTTACGCTGTATGAC ACCATATCCTACCATGTATACCTTTAG
AGATACTTATGATTTTCC TATGTTTAGGCTGTATGAC ACCATATCCTACCATGTATACCTTTAG
16# AGATACTTACGATTTTCC TATGTTTACGCTGTATGAC ACCATATCCTACCATGTATACCTTTAG
AGATACTTACGATTTTCC TATGTTTACGCTGTATGAC ACCATATCCTACCATGTGTACCTTTAG
c
60%
40%
20%
0%
50% (8/16)
50% (8/16)
0% (0/16)
0% (0/16)
SNP-1 + – + –
SNP-2 + – + –
MUT – + + –

Figure S6

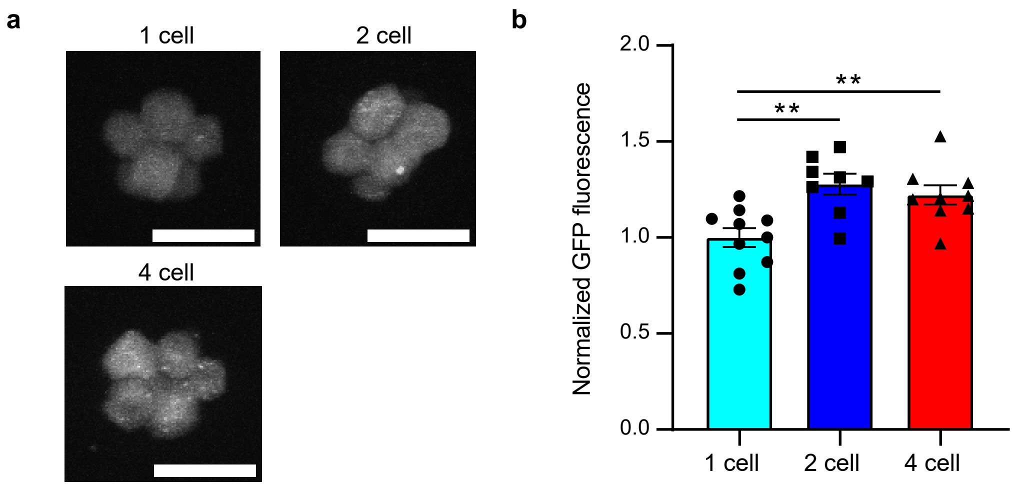
a
1 cell
2 cell
4 cell
b
Normalized GFP fluorescence
2.0
1.5
1.0
0.5
0.0
**
**
1 cell
2 cell
4 cell

Figure S7

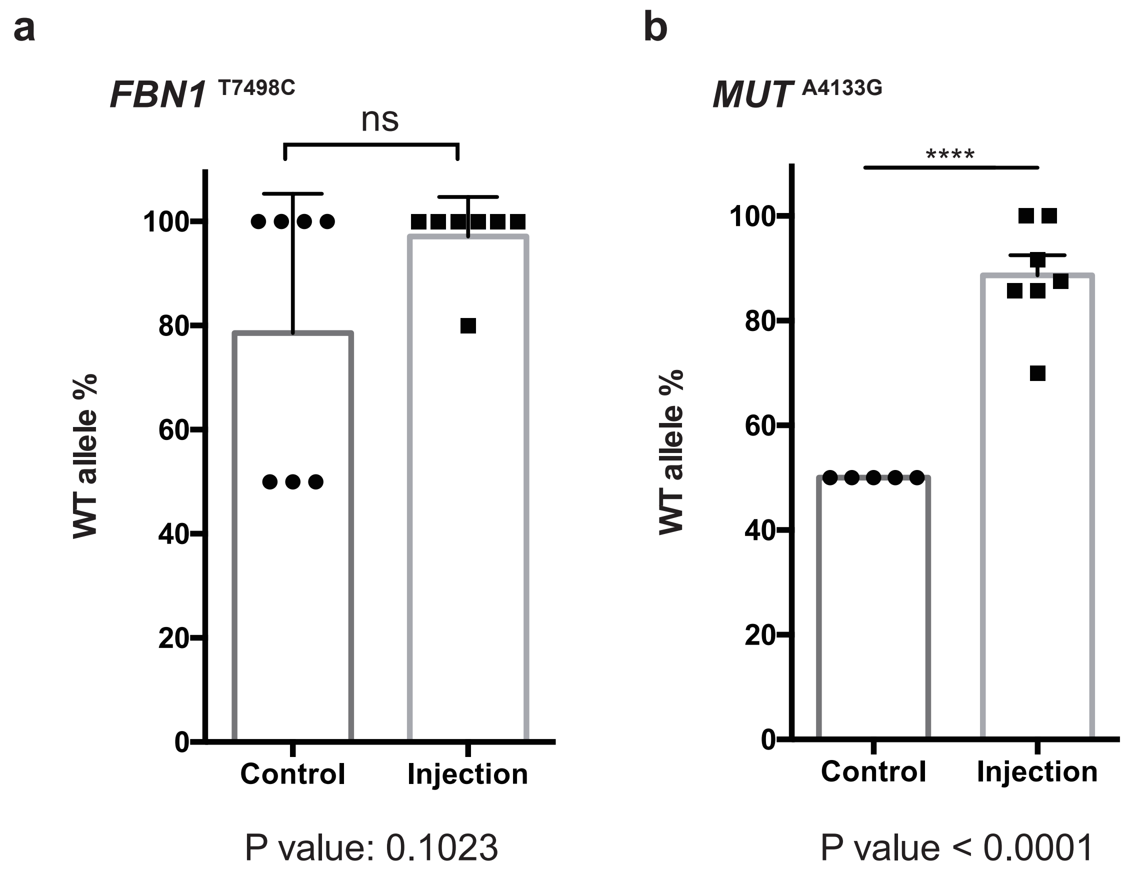
a
FBN1 T7498C
ns
WT allele %
100
80
60
40
20
0
Control
Injection
P value: 0.1023
b
MUT A4133G
****
WT allele %
100
80
60
40
20
0
Control
Injection
P value < 0.0001
