# Supplementary material for: Human cleaving embryos enable robust homozygotic nucleotide substitutions by base editors
Source: Genome Biol. 2019 May 22;20:101. doi: 10.1186/s13059-019-1703-6 (PMC6532253; doi:10.1186/s13059-019-1703-6)
Supplement: Supplementary file 6 — Review history. (DOCX 33 kb) [file 13059_2019_1703_MOESM6_ESM.docx]

Review History

**First round of review**

**Comments to author and authors’ point-by-point responses to the reviewers’ comments:**

**Reviewer 1:**

While the efficiency of DNA targeting by repurposed CRISPR enzymes is quite impressive, the efficiency of introducing precise changes in the DNA sequence (desired indels or point mutations) has been quite low. The base-editing enzyme fused to Cas9 is targeted to specific genomic loci through CRISPR-targeting, wherein the fused base editing enzyme carries out 'the desired point mutation'. Various versions of this base-editing-CRISPR chimeras have been engineered for varying types of point mutations and with varying degree of efficiencies. The low efficiency of these chimeras has always been one of the concerns.

In this manuscript by Zhang et al., the authors have shown that these chimeras can achieve impressively high efficiency in embryos if the mRNAs coding for these chimeras are delivered at the 2-cell or 4-cell embryos stage. The increase in efficiency over the insertion of mRNAs at the 1-cell stage is quite striking, and I believe this useful result should be accepted for the publication. The authors have used this strategy of mRNA insertion at 2-cell/4-cell stage to correct for a disease-causing mutation as well.

The experiments are expertly performed, and the manuscript is quite well-written. But there are some issues which I would like to see addressed before the publication, which are:

1. The manuscript has very little to offer in terms of the reasons behind this increase in base-editing efficiency. The authors mildly speculate that the genome is more compact in the 1-cell stage (compared to 2-cell/4-cells stages), and is, therefore, less amenable to targeting by CRISPR enzymes. While this is fairly decent speculation to make, I believe that there can be other substantial reasons as well, some of which are possible: The differential in the degradation of the mRNAs between 1-cell and 2-cell/4-cell stages which would affect the expression of base-editing chimeras. Can authors perform a blot etc. (or any other suitable assay) to compare the amount of these mRNA between 1-cell and 2-cel/4-cell stages? The amount of RNA from a few embryos may not be sufficient, so maybe RT-PCR can be performed to amplify the mRNAs?

The differential in the translation of base-editing chimeras between 1-cell and 2-cell/4-cell stages. I do not want to make the publication of these results, contingent upon these extra experiments; I leave this decision to authors and the editor(s). Although, I think the addition of any additional input about the reasons behind this increase in base-editing efficiency will make this manuscript quite powerful.

*Response*: *We greatly appreciate your suggestion on the possible reason behind this increase in base-editing efficiency. We have conducted an additional experiment that we injected GFP mRNA into 1-cell, 2-cell and 4-cell embryos and analyzed the intensity of GFP signal 24 hours post injection. Interestingly, we found that the level of GFP fluorescence in 2-cell or 4-cell injected human embryos was significantly higherhan that of 1-cell injected human embryos, implying the differential in the degradation or translation of base-editing mRNAs between 1-cell and 2-cell/4-cell stages. Previous study has shown that a massive RNA degradation event ensues with the first cleavage of zygote and concomitant chromatin change (Miler T. Lee et al., Annu Rev Cell Dev Biol. 2014), consistent with our results regarding different GFP translation and base editing efficiency between one-cell and cleaving embryos. We have added the data (Fig. S5) in the revised manuscript.*

2. Authors must also include a cartoon schematic, in Figure 1, of all the different base-editing-CRISPR chimeras they have used. This addition will increase the readability and clarity of the manuscript. The schematic in Fig.1c of Gaudelli et al. is a good example.

*Response: We agreed with your suggestion and have made the change in the revised manuscript.*

3. I am also confused about the use of the term 'mosaic embryo' in the legend of Fig.2C. Mosaic and Mosaicism refer to the state of the group of cells; wherein some cells are genotypically different from other cells of the group. In this legend, the authors seem to be referring to heterozygous cells as 'mosaic embryo'. Please correct such misleading usage of terms, where they are present in the manuscript.

*Response: We have changed 'homozygous embryo' to 'non-mosaic embryo' in Fig. 2C as you suggested.*

4. The transcription of the RNA (gRNA) with 5'G is significantly higher than that of the ones without it. Therefore, it is usually recommended that the genes containing 3'C (in target strand) or 5'G (in non-target strand) be targeted, because that would allow you to transcribe the gRNA with 5'G. If you need to target such a gene, then you may use a gRNA with extra 5'G (21 nt in the protospacer of crRNA as opposed to 20 nt). But the targeting efficiency of 21 nt gRNA vs 20 nt gRNA is different. So authors must specify clearly in the methods section if 20 nt gRNA was used for all experiments or a combination of 21 and 20 nt.

*Response: We added an extra 5'G in all our gRNAs. We have added this description in our Method section.*

**Reviewer 2**

The manuscript by Meiling Zhang provided a new perspective for genome editing. In comparison to one-cell injection, they found a much higher base editing efficiency by injecting human cleaving 2-cell and 4-cell embryos. Moreover, using this method the homozygotic targeting efficiency is improved and mosaicism is reduced. Undoubtedly, it provides a theoretical basis for human embryo base editing with high efficiency.

I have 3 major concerns:

1 What about the off-target efficiency based on the timing of injections (the number is different in one-cell, 2-cell and 4-cell embryos)? The authors only checked the offtarget effects in 2-cell embryos with one injection.

2 How is editing specificity ensured?

*Response to Q1 and Q2: In this study, we found that 2-cell embryos enable robust homozygotic nucleotide substitutions by base editors and base editing in 2-cell embryos could be potentially applied for gene functional study during early embryonic development in human. Thus, we only checked the off-target effects when embryos edited at 2-cell stage.*

*Recently Lee et al reported that base editing in 1-cell embryos exhibited high fidelity and showed rare off-target effects at predicted off-target sites. Our recent study revealed that base editing in one blastomere of 2-cell embryos also showed no off-target effects at predicted off-target sites. Thus, we think there is no obviously difference on the off-target effects between 1-cell editing and one blastomere of 2-cell editing.*

*To be noted, our group and the other group have reported that cytosine base editors induce numerous off-target mutations in mouse embryos and rice. These mutations were sgRNA-independent and random. Although about 200 SNVs on average would be induced in an embryo treated with BE3, no overlapped SNV was found between any two individual embryos and very few of them would affect gene function. Thus, although current version of BE3 may be not suitable for gene therapies, perturbing gene function by BE3-induced targeted mutation is not likely affected when studying human embryonic development. Certainly, exploring and using high fidelity of base editors for gene manipulation in human embryos is highly desirable in future.*

3 What about the heterogeneity of editing in 2-cell and 4-cell embryos?

*Response: Compared with random indels induced by CRISPR-Cas9 technology, targeted mutations induced by base editors are precise and predictable. Thus, base editors generates far less allelic heterogeneity than conventional Cas9, which makes it more suitable for sophisticated gene functional study in early embryos. Moreover, we revealed that 2-cell editing could substantially reduce mosaicism in contrast with injecting base editors in 1-cell embryos.*

*Though small amount of mosaicism still exist after CRISPR-Cas9 or base editing, it would not affect its application in gene functional study for embryonic development, as demonstrated by Fogarty N. M. E et al. (Fogarty N. M. E et al. Nature. 2017) with conventional Cas9 and Yang G et al. (Yang G et al. Cell Rep. 2019) with base editors.*

*Recently, it has been shown improvement of base editor could further narrow editing windows and improve editing efficiency (Gehrke JM et al., Nat Biotechnol. 2018), which could potentially reduce editing heterogeneity to a much low level.*
